# Characterization of the expression and prognostic value of 14-3-3 isoforms in breast cancer

**DOI:** 10.18632/aging.103919

**Published:** 2020-10-14

**Authors:** Jie Mei, Yan Liu, Rui Xu, Leiyu Hao, An Qin, Chunqiang Chu, Yichao Zhu, Xiao Liu

**Affiliations:** 1 Department of Oncology, Wuxi People’s Hospital Affiliated to Nanjing Medical University, Wuxi 214023, Jiangsu, P.R. China; 2 Department of Physiology, Nanjing Medical University, Nanjing 211166, Jiangsu, P.R. China; 3 Department of General Surgery, Wuxi People’s Hospital Affiliated to Nanjing Medical University, Wuxi 214023, Jiangsu, P.R. China; 4 State Key Laboratory of Reproductive Medicine, Nanjing Medical University, Nanjing 211166, Jiangsu, P.R. China

**Keywords:** 14-3-3, bioinformatic analysis, prognosis, breast cancer

## Abstract

The tyrosine 3-monooxygenase/tryptophan 5-monooxygenase activation proteins (14-3-3) participate in the tumorigenesis and progression of numerous malignances, but their precise prognostic values in breast cancer (BrCa) remain unknown. Here, we investigated the expression profiles and prognostic roles of 14-3-3 isoforms by employing multiple online databases. The transcriptional levels of most 14-3-3 isoforms in BrCa tissues were significantly higher than those in normal tissues. High mRNA expression of 14-3-3 beta/sigma/theta/zeta was significantly associated with poor overall survival (OS) in BrCa patients, while high mRNA expression of 14-3-3 epsilon was notably related to favorable OS. High mRNA expression of 14-3-3 beta/gamma/sigma/theta/zeta was significantly associated with poor relapse-free survival (RFS) in BrCa patients. A high mutation rate of 14-3-3 was determined to be associated with poor clinical outcomes. In addition, 14-3-3 expression was correlated with the infiltration of specific immune cells types. Analysis of the breast-specific protein-protein interaction (PPI) network suggested that 14-3-3 proteins were involved in several potential oncogenic mechanisms in BrCa. Finally, we performed experimentally validated their oncogenic roles in BrCa. Overall, our findings systematically elucidate the expression and distinct prognostic value of 14-3-3 isoforms in BrCa, which may provide potential therapeutic targets and prognostic biomarkers for BrCa.

## INTRODUCTION

Breast cancer (BrCa) is the most frequently occurring cancer in women worldwide [1]. The global incidence of BrCa has been continuously increasing since the late 1970s. In recent years, the incidence rate of increase in China has been higher than that in other countries with high BrCa incidence [2]. According to the latest statistics in China, the annual incidence of BrCa is approximately 272,400 cases [2]. With the development of BrCa screening programs and the progress of comprehensive treatment strategies, the mortality of BrCa has shown a downtrend. Due to the heterogeneity of malignant tumors, the prognosis of specific individuals is varies [3]. Therefore, it is still of great significance for clinicians and scholars to further explore the carcinogenesis of BrCa and provide more applicable prognostic evaluation strategies.

Tyrosine 3-monooxygenase/tryptophan 5-monooxygenase activation proteins (14-3-3), also known as YWHAs, were first reported to exist in mammalian brain tissues [4]. In lactating animals, the 14-3-3 family is a ubiquitous and highly conserved acidic protein family consisting of 7 isoforms (14-3-3 beta, 14-3-3 gamma, 14-3-3 epsilon, 14-3-3 zeta, 14-3-3 eta, 14-3-3 theta, 14-3-3 sigma). 14-3-3 can interact with phosphorylation-dependent proteins and initiate and/or maintain DNA damage monitoring points during the cell cycle by activating mitogen-activated protein kinase (MAPK). 14-3-3 proteins are crucial regulators in coordinating integrin signaling pathways, subsequently inhibiting apoptosis, maintaining cytoskeleton dynamics and promoting cell growth [5]. In recent years, certain studies have indicated that 14-3-3 plays important and complex roles in the initiation and development of human cancers [6–8]. However, the precise prognostic value of 14-3-3 isoforms have not been fully characterized in BrCa.

Recently, bioinformatics approaches have been applied to identify genomic profiles among tumor survivors and develop a mass of cancer-related biomarkers for clinical application [9–11]. For example, our previous research successfully predicted five crucial metastasis-associated genes (TPX2, KIF2C, CDCA8, BUB1B and CCNA2) as promising biomarkers to assess the risk of distant metastasis in BrCa by applying an integrated bioinformatics strategy [12]. We also observed the exact prognostic role of dishevelled associated activator of morphogenesis 1 (DAAM1) in BrCa using Kaplan-Meier plotter, an integrated database containing gene expression profiles and survival data [13], and *in vitro* and *in vivo* assays further validated its critical function [14, 15]. Undoubtedly, bioinformatics-assisted research strategies contribute to a better understanding of BrCa progression and efficient identification of biomarkers.

In this research, we compared the transcriptional levels and prognostic impacts of 14-3-3 isoforms in BrCa using multiple online databases. The cBioPortal database was applied to analyze the genetic alterations of 14-3-3 and their prognostic impacts and potential oncogenic mechanisms. The association between 14-3-3 expression and infiltration levels of immune cells was also assessed. A breast-specific protein-protein interaction (PPI) network was established to speculate the mechanisms of 14-3-3 affecting BrCa development. Finally, we performed pathological and cellular experiments to validate the oncogenic role of 14-3-3 zeta in BrCa tissues and cells. Overall, our research preliminarily and systematically characterized the expression and prognostic impacts of 14-3-3 isoforms in BrCa and suggested that the determination of 14-3-3 expression levels in BrCa patients may be promising indicators for prognostic evaluation.

## RESULTS

### Transcriptional levels of 14-3-3 isoforms across different cancers

**Figure 1:**
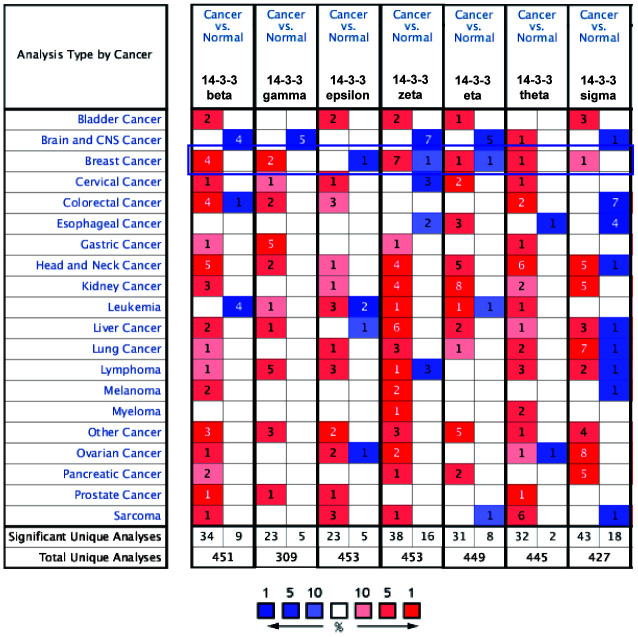
**Transcript levels of 14-3-3 in different types of cancer.** Dysregulation of 14-3-3 isoforms was observed in various cancers. Threshold settings: p-value: 10e-04; fold change: 1.5; gene rank: top 10%. Red represents upregulation, and blue represents downregulation. The numbers in the colored cell represent the numbers of datasets meeting the thresholds.

To obtain a pan-cancer view of 14-3-3 expression, the dysregulation of 14-3-3 isoform transcripts was identified in 20 different types of human cancers in the Oncomine database. 14-3-3 isoforms might act as either tumor promoters or suppressors in various cancers. A total of 19 analyses met the thresholds for 14-3-3 in BrCa (Figure 1). To further determine the expression levels of 14-3-3 genes in BrCa, the data corresponding to the 7 genes with regard to BrCa tissue number, normal tissue number, fold-change, t-test T value, p-value and date source were summarized (Table 1). Among all 14-3-3 isoforms, 14-3-3 beta, 14-3-3 gamma, 14-3-3 theta and 14-3-3 sigma were markedly overexpressed in BrCa tissues compared to normal controls. In addition, 14-3-3 epsilon was downregulated in BrCa, and the other 14-3-3 isoforms exhibited the opposite expression pattern. Overall, we found that the expression of 14-3-3 isoforms varied in BrCa tissues, which indicated that more studies should be conducted to further confirm the abnormal expression of 14-3-3 isoforms.

**Table 1:** The transcriptional levels of 14-3-3 isoforms between BrCa and normal tissues.

**14-3-3 isoforms**	**BrCa tissues**	**BrCa samples**	**Normal samples**	**Fold-change**	**t-Test**	**p-value**	**PMID or data source**
14-3-3 beta	Invasive Ductal Breast Carcinoma	9	14	1.861	5.449	3.70E-05	19187537
	Male Breast Carcinoma	3	61	1.82	9.081	7.59E-05	TCGA
	Invasive Ductal Breast Carcinoma	389	61	1.573	10.686	6.04E-18	TCGA
	Ductal Breast Carcinoma	40	7	1.788	5.434	8.59E-05	16473279
14-3-3 gamma	Intraductal Cribriform Breast Adenocarcinoma	3	61	2.801	8.675	4.29E-12	TCGA
	Ductal Breast Carcinoma in Situ	9	14	1.978	5.005	9.47E-05	19187537
14-3-3 epsilon	Invasive Breast Carcinoma Stroma	53	6	-14.768	-23.246	9.54E-28	18438415
14-3-3 zeta	Ductal Breast Carcinoma	40	7	1.774	7.768	7.77E-09	16473279
	Ductal Breast Carcinoma in Situ	9	14	2.126	5.815	3.70E-05	19187537
	Intraductal Cribriform Breast Adenocarcinoma	3	61	1.766	4.923	7.07E-06	TCGA
	Invasive Ductal Breast Carcinoma	389	61	1.799	14.658	6.44E-31	TCGA
	Invasive Breast Carcinoma	76	61	1.717	7.303	1.24E-11	TCGA
	Mucinous Breast Carcinoma	46	144	1.784	8.645	9.82E-12	22522925
	Invasive Ductal and Invasive Lobular Breast Carcinoma	90	144	1.522	10.191	5.79E-18	22522925
	Invasive Breast Carcinoma Stroma	53	6	-17.007	-20.802	1.83E-21	18438415
14-3-3 eta	Mucinous Breast Carcinoma	4	61	1.575	6.141	3.80E-05	TCGA
	Invasive Breast Carcinoma Stroma	53	6	-5.53	-14.306	5.95E-20	18438415
14-3-3 theta	Ductal Breast Carcinoma	40	7	1.612	7.038	4.93E-09	16473279
14-3-3 sigma	Invasive Ductal and Invasive Lobular Breast Carcinoma	90	144	1.591	8.521	1.71E-15	22522925

### Transcriptional levels of 14-3-3 isoforms in BrCa samples.

**Figure 2:**
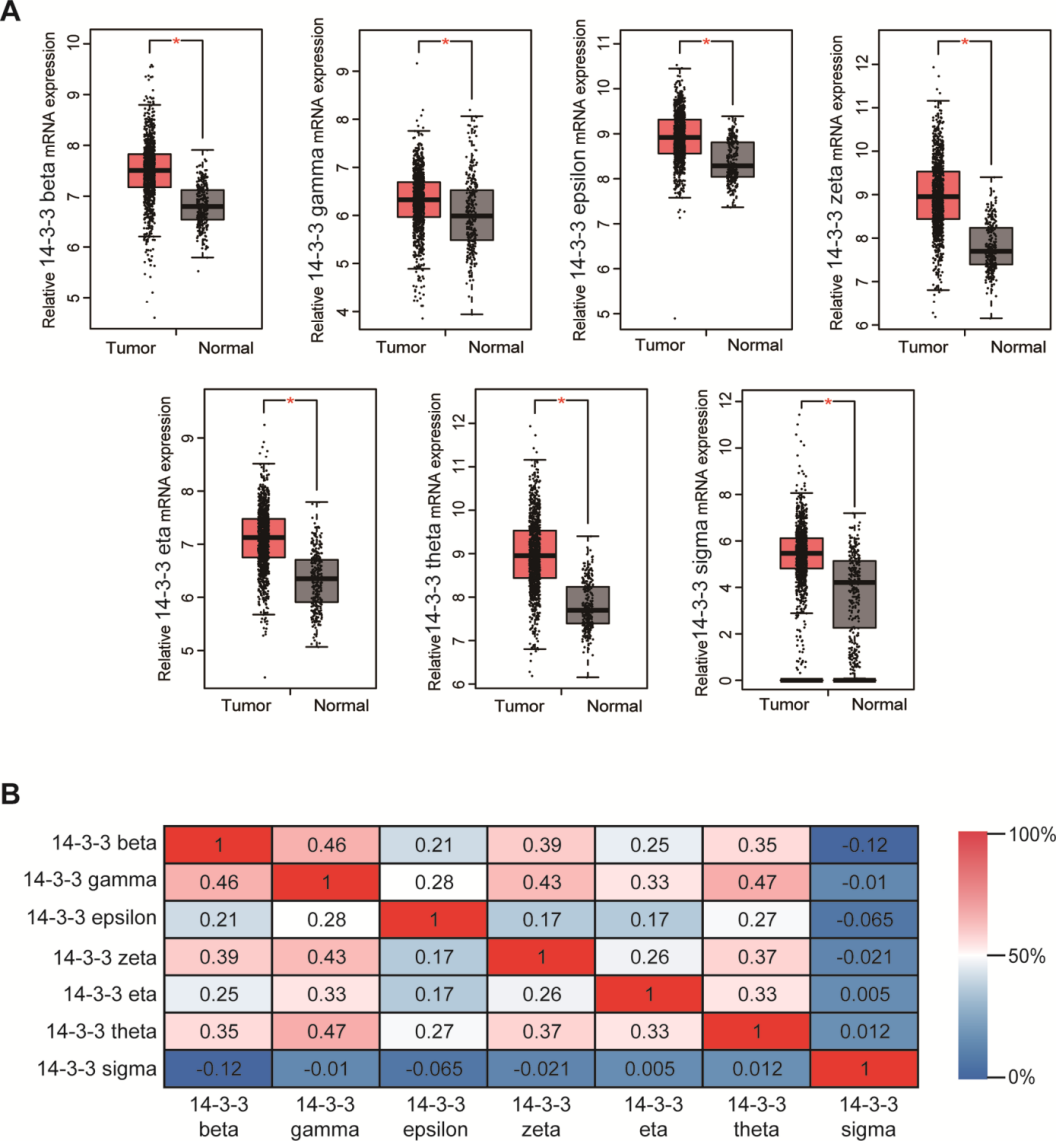
**Transcriptional levels of 14-3-3 in BrCa tissues.** (**A**) Box plots derived from gene expression data in GEPIA comparing the expression of 14-3-3 in BrCa and normal tissues. The p-value was set up at 0.05. (**B**) The Pearson correlation coefficients between 14-3-3 isoforms.

For the purpose of exploring the therapeutic potential of 14-3-3 members in BrCa patients, the differential transcriptional levels of 14-3-3 isoforms between BrCa and normal breast tissues were further determined by GEPIA. As shown in Figure 2A, the transcriptional levels of 14-3-3 beta (P<0.05), 14-3-3 gamma (P<0.001), 14-3-3 epsilon (P<0.05), 14-3-3 zeta (P<0.05), 14-3-3 eta (P<0.05), 14-3-3 theta (P<0.05), and 14-3-3 sigma (P<0.05) were all significantly upregulated in BrCa tissues compared with paracancerous tissues. Moreover, we assessed the correlations of 14-3-3 isoforms with each other by analyzing their mRNA expression *via* GEPIA and performing Pearson’s correction. The results indicated significant and positive correlations between 14-3-3 isoforms, while 14-3-3 sigma exhibited quiet low correlations with the six other 14-3-3 isoforms (Figure 2B). Overall, the correlation analysis showed that 14-3-3 isoforms, except 14-3-3 sigma, had high coexpression features and might share a similar molecular function.

### Association of mRNA expression of 14-3-3 isoforms with the clinical characteristics of BrCa patients.

**Figure 3:**
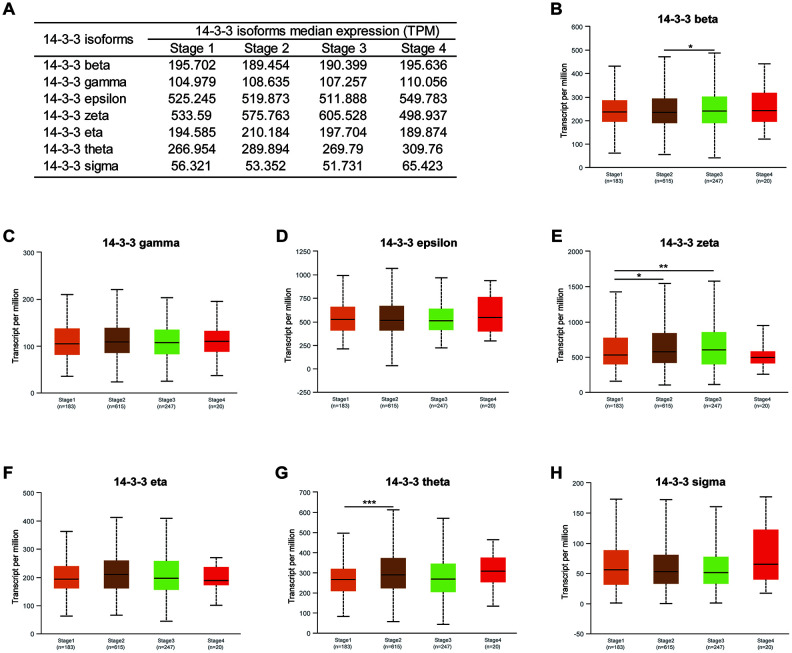
**Association of mRNA expression of 14-3-3 isoforms with clinical stages of BrCa.** (**A**) Summarization of transcriptional expression of 14-3-3 isoforms in different clinical stages. (**B**, **E**, **G**) Expression levels of 14-3-3 beta, 14-3-3 zeta and 14-3-3 theta were related to clinical stages. (**C**, **D**, **F**, **H**) Expression levels of 14-3-3 gamma, 14-3-3 epsilon, 14-3-3 eta and 14-3-3 sigma were not associated with clinical stages.

We next analyzed the association between the mRNA expression of 14-3-3 isoforms and the clinical characteristics of BrCa patients by UALCAN, including patients’ clinical stages and molecular subtypes. The mRNA expression of 14-3-3 isoforms in different clinical stages is summarized in Figure 3A. 14-3-3 beta, 14-3-3 zeta, and 14-3-3 theta were significantly correlated with advanced clinical stage (Figure 3B, 3E, 3G), while other 14-3-3 isoforms were not obviously associated with different clinical stages (Figure 3C, 3D, 3F, 3H).

**Figure 4:**
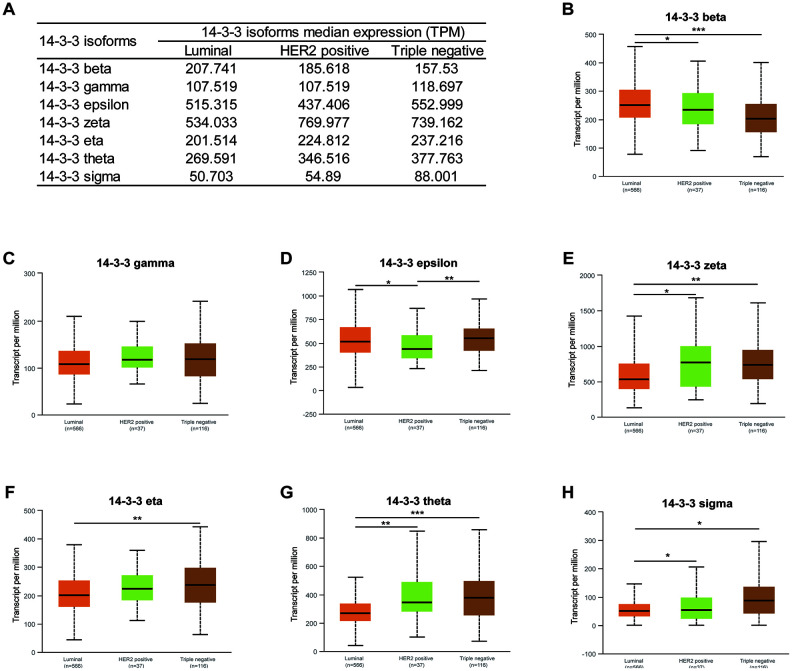
**Association of mRNA expression of 14-3-3 isoforms with molecular subtypes of BrCa.** (**A**) Summarization of transcriptional expression of 14-3-3 isoforms in different molecular subtypes. (**B**, **D**, **E**, **F**, **G**, **H**) Expression levels of 14-3-3 beta, 14-3-3 epsilon, 14-3-3 zeta, 14-3-3 eta, 14-3-3 theta and 14-3-3 sigma were related to molecular subtypes. (**C**) The expression level of 14-3-3 gamma was not associated with molecular subtype.

Moreover, the mRNA expression levels of six 14-3-3 isoforms were related to molecular subtype. The transcriptional expression of 14-3-3 isoforms in different molecular subtypes is summarized in Figure 4A. The highest expression levels of 14-3-3 epsilon, 14-3-3 eta, 14-3-3 theta, and 14-3-3 sigma were found in triple-negative breast cancer tissues (Figure 4D, 4F, 4G, 4H). In addition, 14-3-3 beta was enriched in luminal breast cancer tissues (Figure 4B), and 14-3-3 zeta was enriched in HER2-positive breast cancer tissues (Figure 4E). However, the expression of 14-3-3 gamma was not obviously related to molecular subtypes of BrCa (Figure 4C).

### Prognostic value of 14-3-3 isoform mRNA expression in BrCa samples.

**Figure 5:**
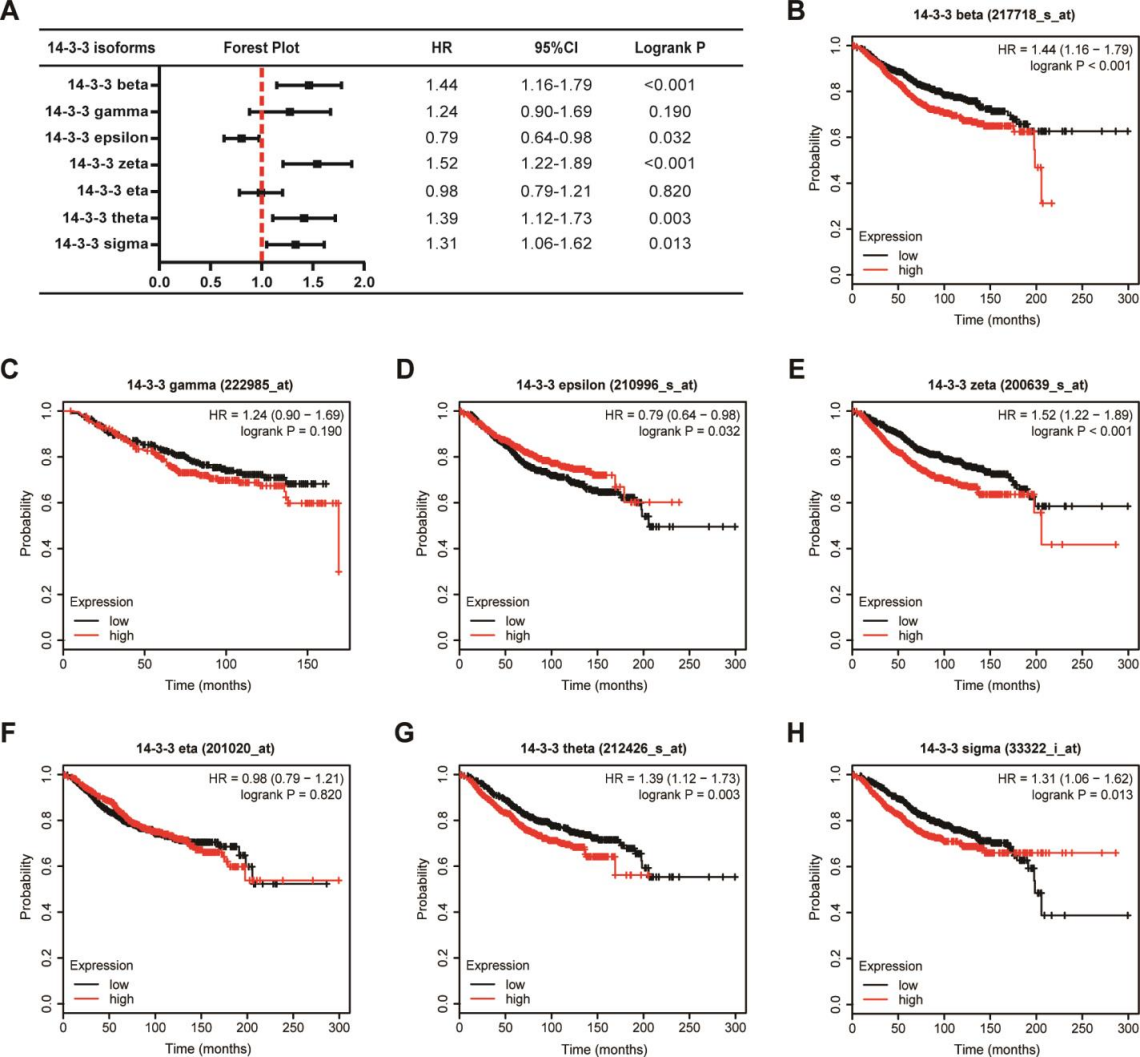
**Prognostic value of 14-3-3 isoforms in BrCa patients (OS).** (**A**) Forest map exhibiting the prognostic value of 14-3-3 isoforms in predicting OS. OS curves were plotted to evaluate the prognostic value of 14-3-3 isoform mRNA expression. (**B**) 14-3-3 beta, (**C**) 14-3-3 gamma, (**D**) 14-3-3 epsilon, (**E**) 14-3-3 zeta, (**F**) 14-3-3 eta, (**G**) 14-3-3 theta, (**H**) 14-3-3 sigma.

Further, we applied Kaplan-Meier plotter to assess the prognostic value of 14-3-3 members in BrCa (Figure 5A). As shown in Figure 5, high mRNA expression levels of 14-33 beta (HR=1.44, 95%CI: 1.16-1.79, P<0.001, Figure 5B), 14-3-3 zeta (HR=1.52, 95%CI: 1.22-1.89, P<0.001, Figure 5E), 14-3-3 theta (HR=1.39, 95%CI: 1.12-1.73, P=0.003, Figure 5G), and 14-3-3 sigma (HR=1.31, 95%CI: 1.06-1.62, P<0.001, Figure 5H) were significantly associated with poor overall survival (OS) of BrCa patients, while high mRNA expression of 14-3-3 epsilon was notably related to favorable OS of BrCa patients (HR=0.79, 95%CI: 0.64-0.98, P=0.032, Figure 5D). However, the mRNA expression of other 14-3-3 isoforms was not associated with the prognosis of BrCa patients (Figure 5C, 5F).

**Figure 6:**
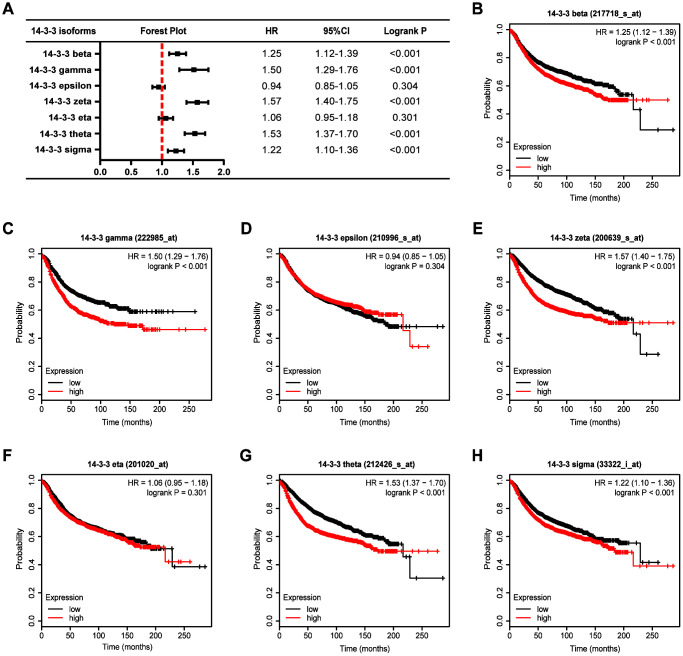
**Prognostic value of 14-3-3 isoforms in BrCa patients (RFS).** (**A**) Forest map exhibiting the prognostic value of 14-3-3 isoforms in predicting RFS. RFS curves were plotted to evaluate the prognostic value of 14-3-3 isoform mRNA expression. (**B**) 14-3-3 beta, (**C**) 14-3-3 gamma, (**D**) 14-3-3 epsilon, (**E**) 14-3-3 zeta, (**F**) 14-3-3 eta, (**G**) 14-3-3 theta, (**H**) 14-3-3 sigma.

We next analyzed the associations between 14-3-3 isoform mRNA expression levels and relapse-free survival (RFS) in BrCa patients (Figure 6A) and found that high mRNA expression levels of 14-3-3 beta (HR=1.25, 95%CI: 1.12-1.39, P<0.001, Figure 6B), 14-3-3 gamma (HR=1.50, 95%CI: 1.29-1.76, P<0.001, Figure 6C), 14-3-3 zeta (HR=1.57, 95%CI: 1.40-1.75, P<0.001, Figure 6G), 14-3-3 theta (HR=1.53, 95%CI: 1.37-1.70, P<0.001, Figure 6E), and 14-3-3 sigma (HR=1.22, 95%CI: 1.10-1.36, P<0.001, Figure 6H) were significantly associated with shorter RFS of BrCa patients, while the expression levels of 14-3-3 epsilon and 14-3-3 eta were not related to outcomes of BrCa patients (Figure 6D, 6F). Overall, these findings indicated that the mRNA expression levels of 14-3-3 beta, 14-3-3 zeta, 14-3-3 theta, and 14-3-3 sigma were markedly correlated with BrCa patients’ OS and RFS, indicating that they might be considered promising indicators for predicting the survival times of BrCa patients.

### Genetic alterations of 14-3-3 isoforms in BrCa samples.

**Figure 7:**
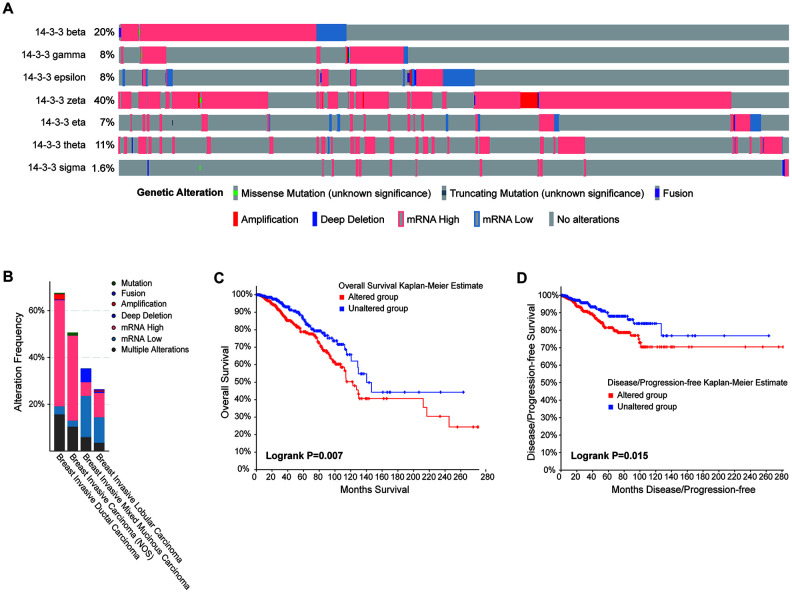
**Correlation between the genetic alterations of 14-3-3 and prognosis.** (**A**) Oncoprint in the cBioPortal database showed the proportion and distribution of specimens with genetic alterations of 14-3-3 isoforms. (**B**) Box plot showing the proportion and distribution of 14-3-3 isoforms in BrCa subtypes. (**C**) OS curves were plotted to evaluate the prognostic impacts of 14-3-3 isoform mutations. (**D**) DFS curves were plotted to evaluate the prognostic impacts of 14-3-3 isoform mutations.

Genomic mutations are closely associated with the oncogenesis and progression of cancers. To obtain comprehensive insight into the genomic mutation of 14-3-3 isoforms in BrCa, the cBioPortal database was applied. As exhibited in Figure 7A, high proportions and multiple types of alterations of 14-3-3 were found in BrCa patients, with the most common alteration being high mRNA expression of 14-3-3. In addition, 14-3-3 zeta had a high mutation frequency in the BrCa sample, which added up to 40% (Figure 7A). 14-3-3 had a relatively high mutation frequency in all BrCa subtypes and showed a high mutation frequency in majority of the breast invasive ductal carcinomas (Figure 7B). Furthermore, Kaplan-Meier analysis revealed that patients with genetic alterations in 14-3-3 had worse OS (P=0.007, Figure 7C) and disease-free survival (DFS) than those with alterations (P=0.015, Figure 7D).

**Figure 8:**
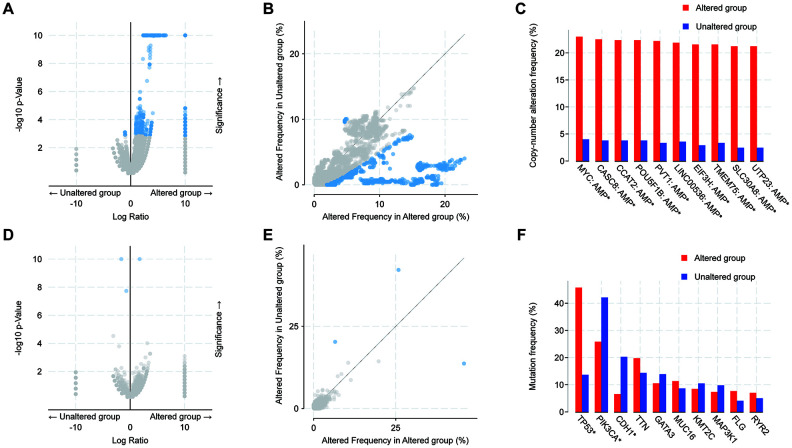
**Analysis of global genes associated with mutations in 14-3-3.** (**A**, **B**) Scatter and volcano plots exhibiting genes associated with alterations in 14-3-3 CNA frequency. (**C**) Box blot representing the 10 most frequently altered genes. Amp: amplification. (**D**, **E**) Scatter and volcano plots exhibiting genes associated with mutations altered frequency in 14-3-3. (**F**) Box blot representing the 10 most frequently altered genes; only 3 genes exhibited significant differences.

Next, we explored the oncogenic mechanisms of 14-3-3 mutations in BrCa. We compared the gene enrichment differences in altered and unaltered BrCa cohorts based on common mutations and copy number alterations(CNAs), respectively. The results showed that the CNA (amplification and deep deletion) frequency of 14-3-3 had notable associations with that in thousands of genes (Figure 8A, 8B). The 10 most frequently altered genes were MYC, CASC8, CCAT2, POU5F18, PVT1, LINC00536, EIF3H, TMEM75, SLC30A8, and UTP23 (Figure 8C). In addition, altered 14-3-3 mutation (missense mutation, truncating mutation and fusion) frequency had few associations with that in other genes (Figure 8D, 8E). Only 3 genes exhibited significant differences, namely, TP53, PIK3CA, and CDH1 (Figure 8F). To conclude, the results suggested that 14-3-3 mutations showed close associations with the tumorigenesis and prognosis of BrCa.

### Association between TIICs and 14-3-3 members

**Figure 9:**
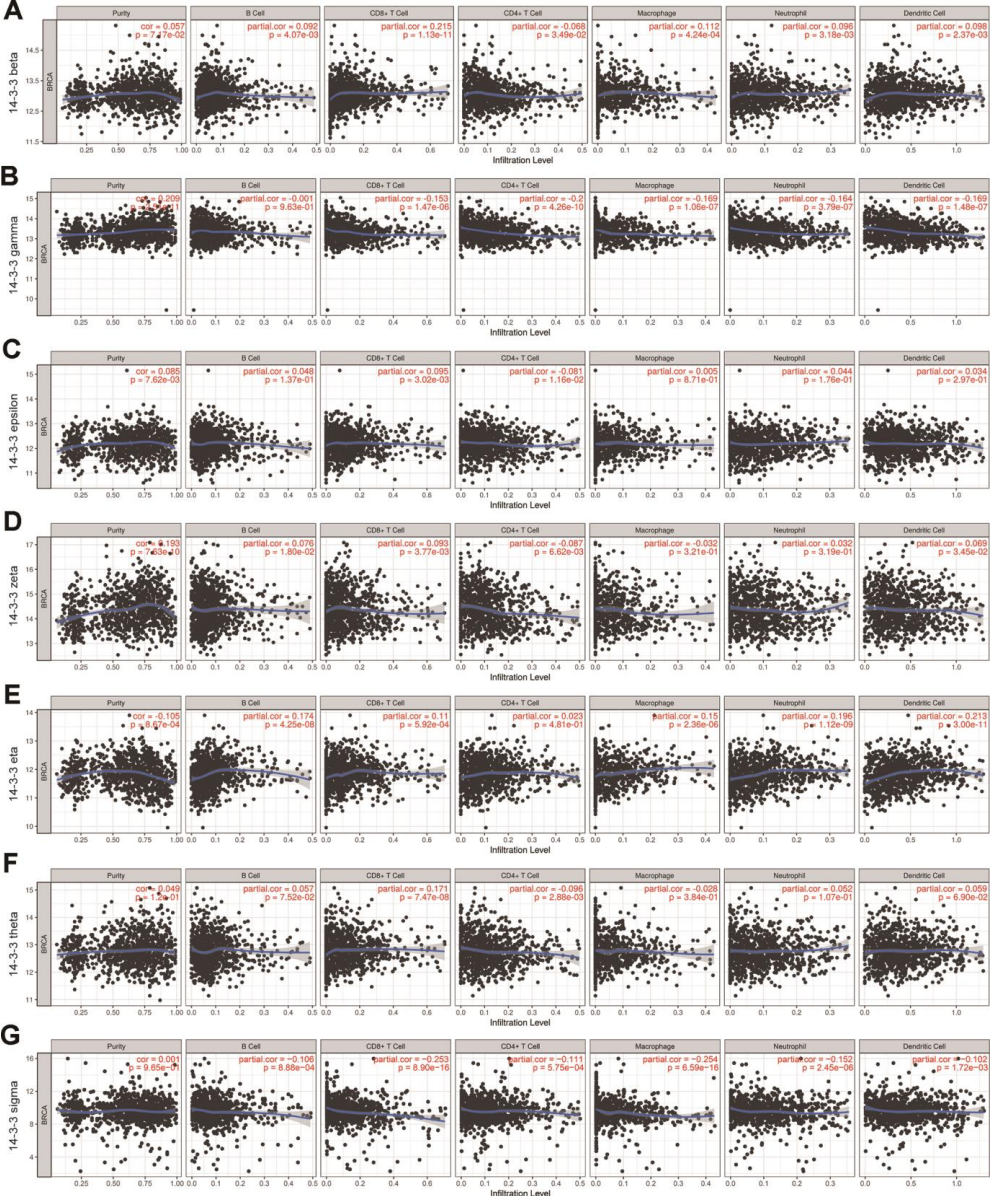
**Correlation of TIICs and 14-3-3.** Tumor purity is shown in the left panel. The correlation of seven 14-3-3 isoforms and TIICs (B cells, CD4+ T cells, CD8+ T cells, neutrophils, macrophages and dendritic cell types) is displayed. (**A**) 14-3-3 beta, (**B**) 14-3-3 gamma, (**C**) 14-3-3 epsilon, (**D**) 14-3-3 zeta, (**E**) 14-3-3 eta, (**F**) 14-3-3 theta, (**G**) 14-3-3 sigma.

Given the distinct prognostic impacts of 14-3-3, the potential immunological correlation of 14-3-3 members and tumor-infiltrating immune cells (TIICs) was assessed when the correlation coefficient was greater than 0.2. Among the 14-3-3 isoforms, only 14-3-3 gamma had a close association with tumor purity (cor=0.209, P=2.51e-11, Figure 9B). Intriguingly, the highest correlation with TIICs was identified between 14-3-3 sigma and macrophages (cor=-0.254, P=6.59e-16, Figure 9G). Moreover, the correlations between 14-3-3 beta and CD8+ T cells (cor=0.215, P=1.13e-11, Figure 9A), 14-3-3 gamma and CD4+ T cells (cor=0.200, P=4.26e-10, Figure 9B), 14-3-3 eta and dendritic cells (cor=0.213, P=3.00e-11, Figure 9E) and 14-3-3 sigma and CD8+ T cells (cor=-0.253, P=8.90e-16, Figure 9G) were comparably notable. Overall, these findings imply that 14-3-3 expression might be a latent indicator of the abundance of specific TIICs.

### Construction of breast-specific PPI and enrichment analysis of 14-3-3

**Figure 10:**
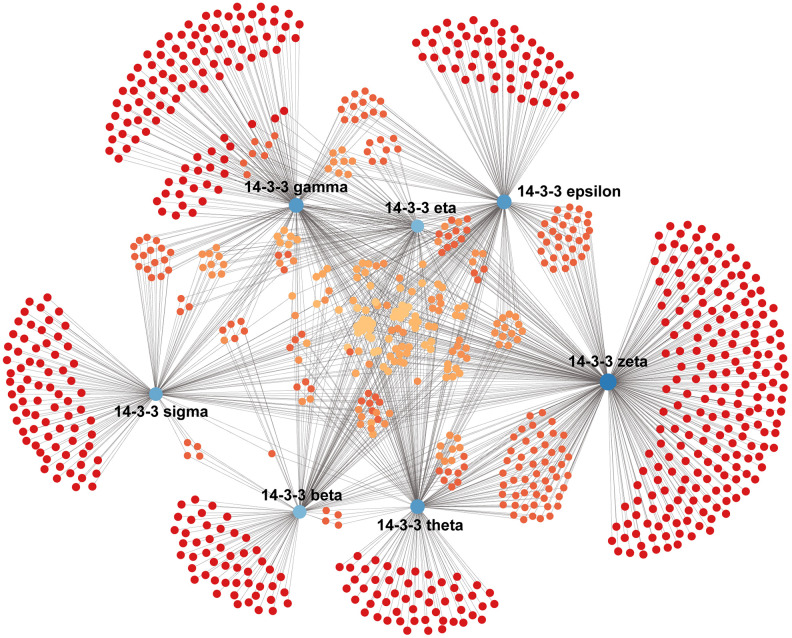
**Construction of the breast-specific PPI network of 14-3-3.** The PPI network of 14-3-3 was constructed on the NetworkAnalyst website. Blue nodes represent 14-3-3 isoforms, and red and orange nodes represent crucial interactors in breast mammary tissues.

**Figure 11:**
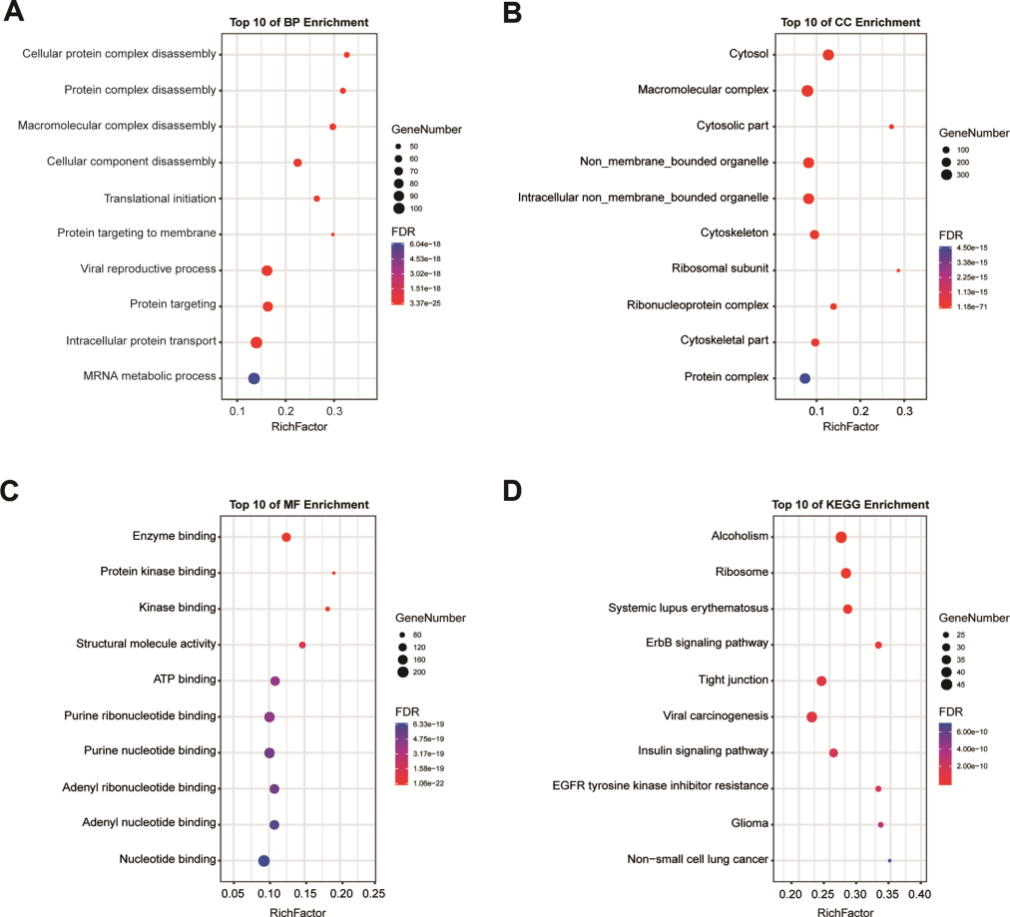
**GO and KEGG pathway enrichment analysis.** Enrichment analysis was performed to preliminarily explain the potential mechanisms of 14-3-3 isoforms and their interactors. (**A**) Biological process, (**B**) cellular component, (**C**) molecular function and (**D**) KEGG pathway analyses. The size of each circle indicates the counting number on each term, while the color represents the p-value of the enrichment analysis.

We constructed a breast-specific PPI for 14-3-3 isoforms by using NetworkAnalyst to explore their potential mechanisms in the tumorigenesis of BrCa. The PPI network is shown in Figure 10. A total of 890 crucial nodes were constructed (Supplementary Table 1). Furthermore, we also conducted Gene Ontology (GO) and Kyoto Encyclopedia of Genes and Genomes (KEGG) analyses to gain insight into their potential functions. GO enrichment analysis predicted the functional roles of genes of interest on the basis of three aspects, including biological processes, cellular components, and molecular functions. A mass of meaningful terms was identified, and we retained the top 10 terms of each analysis to plot the bubble chart (Figure 11). As exhibited in KEGG terms, high ranking terms, such as alcoholism, ribosome, systemic lupus erythematosus, ErbB signaling pathway, tight junction, viral carcinogenesis, *etc.* had notable impacts on the carcinogenesis of human cancers (Figure 11). Here, GO and KEGG analyses enriched the understanding of how 14-3-3 participates in BrCa oncogenesis and progression.

### Experimental validation of the expression and role of 14-3-3 zeta in BrCa

**Figure 12:**
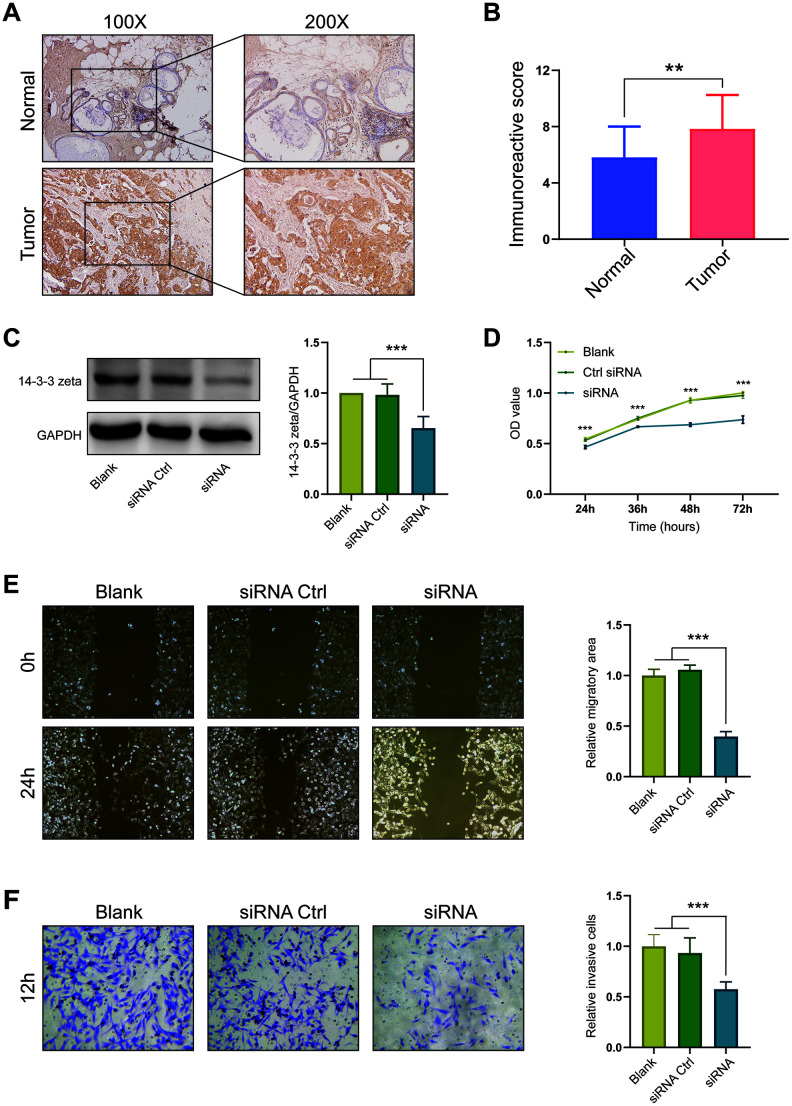
**Expression and oncogenic role of 14-3-3 zeta in BrCa.** (**A**) Representative microphotographs revealing 14-3-3 zeta expression in BrCa and normal tissues using IHC staining. (**B**) T-test analysis of 14-3-3 zeta expression in BrCa and normal tissues. (**C**) 14-3-3 zeta protein downregulation after transfection of 14-3-3 zeta-specific siRNA. The 14-3-3 zeta-specific siRNA suppressed the (**D**) viability, (**E**) migration, and (**F**) invasion of MDA-MB-231 cells.

By a series of bioinformatics analyses of 14-3-3 isoforms, we found that 14-3-3 zeta showed the most significant expression difference between BrCa and control samples and had satisfactory prognostic value. Thus, we performed experiments to validate the expression and role of 14-3-3 zeta in BrCa. Through immunohistochemistry (IHC) staining, we found that the 14-3-3 zeta was highly expressed in BrCa compared with normal tissues, which was in accordance with the findings from bioinformatics analysis (Figure 12A, 12B). Next, we used a siRNA to knockdown 14-3-3 zeta and determined the malignant phenotypes of BrCa cells. First, we examined the knockdown efficiency of the siRNA targeting 14-3-3 zeta by Western blotting (Figure 12C). Then, we found that BrCa cells with 14-3-3 zeta knockdown exhibited reduced viability, migration, and invasion capacities (Figure 12D, 12E, 12F). Overall, all these findings confirmed the oncogenic role of 14-3-3 zeta in BrCa.

## DISCUSSION

Dysregulation of 14-3-3 isoforms has been observed in multiple cancers. Although the roles of 14-3-3 isoforms in the tumorigenesis and progression of several tumors have been partially confirmed [8, 16, 17], a systematic bioinformatic analysis of 14-3-3 has been conducted in BrCa. This study is the first to systemically investigate the expression and prognostic impacts of 14-3-3 isoforms in BrCa. Our findings will bring new insights supporting the improvement of the treatment design and prognosis evaluation for BrCa patients.

14-3-3 beta is significantly overexpressed in several cancers, such as osteosarcoma [18] and hepatocellular carcinoma [19]. 14-3-3 beta has oncogenic potential in BrCa as it directly binds to ERα and activates the transcriptional activity of ERα [20]. However, its expression and prognostic impact in BrCa have not been studied. In our research, we found that 14-3-3 beta is upregulated in BrCa tissues and that 14-3-3 beta overexpression is associated with poor OS and RFS and CD8+ T cell infiltration.

14-3-3 gamma is a tumor activator in BrCa and is associated with tumor progression in BrCa [21]. Hiraoka et al. reported that 14-3-3 gamma is specifically located in the pseudopodia of MDA-MB-231 BrCa cells and that the downregulation of 14-3-3 gamma can prevent the invasion and metastasis of BrCa [22]. In addition, YWHAG was identified as a direct target of miR-181b-3p, the downregulation of which induces Snail stabilization and epithelial-mesenchymal transition (EMT) phenotypes in BrCa cells [23]. In this report, Oncomine and GEPIA dataset analyses revealed that the E2F1 transcript was overexpressed in BrCa compared with normal breast tissues. Based on Kaplan-Meier Plotter, we evaluated the prognostic impact of 14-3-3 gamma in patients. High 14-3-3 gamma mRNA expression was notably related to poor OS and RFS in BrCa patients. In addition, 14-3-3 gamma had a close association with tumor purity and CD8+ T cell infiltration.

There is little research about the role of 14-3-3 epsilon in BrCa. Li et al. reported that the expression of the 14-3-3 epsilon is upregulated in a highly metastatic variant of parental MDA-MB-435 BrCa cells by mass spectrometry, revealing that the 14-3-3 epsilon may play a potential role in tumor metastasis [24]. In addition, knockdown of 14-3-3 epsilon expression reduced the expression of Snail and Twist in BrCa cells [25]. In our study, 14-3-3 epsilon exhibited contradictory expression status between the Oncomine and GEPIA databases. Interestingly, the prognostic value of 14-3-3 epsilon was also obscure. Overexpression of 14-3-3 epsilon was associated with favorable OS but had no relation to RFS. Moreover, the expression of 14-3-3 epsilon was not correlated with tumor purity or immune cell infiltration.

14-3-3 zeta is overexpressed in all BrCa tissues of different stages and grades compared with normal breast tissues [26]. In addition, overexpression of 14-3-3 zeta is an independent prognostic indicator for reduced DFS, and knockdown of 14-3-3 zeta expression by siRNA in BrCa cells effectively reduces tumor growth *in vitro* and *in vivo* [27]. 14-3-3 zeta also promotes resistance to paclitaxel in BrCa. Exogenous miR-451 significantly suppresses β-catenin expression by directly targeting 14-3-3 zeta and subsequently reverses resistance to paclitaxel [28]. Moreover, 14-3-3 zeta can turn TGF-β from a tumor suppressor to a metastatic promoter in BrCa via contextual changes in Smad partners from p53 to Gli2 [17]. In our research, our findings revealed the overexpression and unfavorable prognostic role of 14-3-3 zeta in BrCa, which is consistent with the role of 14-3-3 zeta as a tumor promoter according to previous publications. Furthermore, we also validated the tumor promoter role of 14-3-3 zeta in BrCa using cellular experiments.

The role of 14-3-3 eta in BrCa has not been well defined. Liang et al. revealed that 14-3-3 eta is expressed at significantly higher levels in BrCa tissues than in normal breast tissues [29]. 14-3-3 eta is a partner of gremlin 1, which is overexpressed in multiple cancers, including carcinomas of the lung, ovary, kidney, colon, pancreas and sarcoma. Thus, gremlin 1 and 14-3-3 eta could be promising targets for exploring novel diagnostic and therapeutic strategies against human cancers [30]. In our report, similar to 14-3-3 epsilon, 14-3-3 eta exhibited contradictory expression patterns, and no significant prognostic value was found in BrCa. However, 14-3-3 eta expression was notably associated with the infiltration of dendritic cells in BrCa.

The expression level of 14-3-3 theta is significantly higher in BrCa tissues based on a study containing 216 patients [31]. In addition, knockdown of 14-3-3 theta expression in BrCa cells significantly suppresses migration and invasion *in vitro* and *in vivo* [31]. Based on proteomic identification, Hodgkinson et al. reported the potential role of 14-3-3 theta as a marker for assessing the curative effect of neoadjuvant chemotherapy in BrCa. In our report, the expression of 14-3-3 theta in BrCa tissues was upregulated compared with that in normal breast tissues. We also suggested that 14-3-3 theta expression is significantly associated with poor OS and RFS.

Many studies have well defined the role of 14-3-3 sigma in BrCa. 14-3-3 sigma is usually downregulated in BrCa [32, 33]. Hypermethylation is the main reason for the loss of BrCa expression, and 14-3-3 sigma promoter methylation level has been identified as a new biomarker in blood specimens for BrCa diagnosis [34]. Ji et al. found that the silencing of filamin A inhibits the migration and invasion of BrCa cells by upregulating 14-3-3 sigma, suggesting a tumor suppressive role as well [35]. However, to our surprise, upregulated 14-3-3 sigma expression was observed in our research, and high 14-3-3 sigma expression was significantly correlated with poor OS and RFS in all of the patients with BrCa. In addition, 14-3-3 sigma expression showed a negative relation to the infiltration of macrophages and CD8+ T cells. All our findings seemed inconsistent with the role of 14-3-3 sigma as a tumor suppressor. Ko et al. also challenged the tumor suppressive role of 14-3-3 sigma. They found 14-3-3 sigma is an important poor prognostic biomarker in BrCa, which is consistent with our results [36].

As an important part of this research, we explored the genetic alterations of 14-3-3 isoforms in BrCa tissues. High proportions and multiple types of alterations of 14-3-3 were found in BrCa patients, and high mRNA expression was the most common alteration of 14-3-3. Moreover, Kaplan-Meier analysis revealed that patients with genetic alterations in 14-3-3 had worse OS and DFS than those without genetic alterations, suggesting that not only the expression of 14-3-3 was an important indicator for prognosis but also the joint detection of mutations in 14-3-3 genes was significant for survival prediction in BrCa patients.

14-3-3 proteins regulate certain signaling pathways by phosphorylation-dependent binding with partner proteins [37]. Identification of 14-3-3 isoform-associated networks is essential to exploring the potential mechanism of 14-3-3-mediated BrCa progression. Thus, we constructed a breast-specific PPI network for 14-3-3 by using NetworkAnalyst. Only a small number of shared genes interacted with all 14-3-3 isoforms, and specific 14-3-3 isoforms tended to have different cooperative partners, suggesting various functions of 14-3-3 isoforms in BrCa. Several studies have revealed the different roles of 14-3-3 isoforms in BrCa as well [16], and the PPI networks in our research may provide comprehensive insights for exploring the mechanisms of 14-3-3 isoforms with different roles in BrCa.

In summary, we systemically analyzed the expression and prognostic value of 14-3-3 isoforms in BrCa. Our results revealed that 14-3-3 might be a potential therapeutic target for BrCa therapy and that 14-3-3 beta, 14-3-3 zeta, 14-3-3 theta and 14-3-3 sigma are promising prognostic biomarkers for indicating OS and RFS in BrCa patients. Overall, our research provides systematic insights into the precise prognostic roles and potential oncogenic roles of 14-3-3 in BrCa.

## MATERIALS AND METHODS

### Bioinformatics analysis

### Oncomine database analysis

The Oncomine database (https://www.oncomine.org/resource/login.html) is an online website that allows users to explore gene expressions of interest in largest tumor and normal tissue panels [38]. In this research, Oncomine was used to determine the relative expression levels of the 14-3-3 genes across various cancers. Only analyses regarding gene expression between tumor tissues and normal tissues were reserved for further analysis. Relative parameters for analysis were set as follows: p-value of 10E-04, fold change of 1.5 and threshold for gene rank of all. Data types included both DNA and mRNA subtypes in the present analysis.

### GEPIA database analysis

The GEPIA database (http://gepia.cancer-pku.cn/) is an interactive web based on the TCGA dataset, which utilizes RNA sequencing to conduct gene expression analyses [39]. In the present study, the GEPIA website was used to investigate the expression levels of 14-3-3 in BrCa and normal breast tissue specimens. In addition, analysis of correlations between 14-3-3 expressions was also performed using the browser. The cutoff p-value of the differential levels of 14-3-3 was defined as 0.05.

### Kaplan-Meier plotter analysis

Kaplan-Meier Plotter (http://kmplot.com/analysis/) is an online database containing gene expression profiles and survival data of thousands of cancer patients [13]. The prognostic value of transcriptional 14-3-3 isoforms in BrCa were analyzed using Kaplan-Meier Plotter. The patient cohorts were split according to the median expression of each 14-3-3 isoform. Only the JetSet best probe set was allowed to evaluate 14-3-3 isoform expression. All kinds of survival subtypes, including OS and RFS, were included in this analysis. All cohorts were compared with Kaplan-Meier survival plots. The hazard ratio (HR), 95% confidence interval (95%CI), and logrank p-value were calculated and displayed online.

### cBioPortal database analysis

cBioPortal (http://www.cbioportal.org) is a user-friend, interactive website that offers visualization and analysis of large-scale cancer genomics datasets [40, 41]. To explore the 14-3-3 mutations in BrCa and their prognostic impacts, the cBioPortal database was applied. In addition, enrichment analysis of global genes associated with 14-3-3 mutations was also inspected by cBioPortal. The top 10 genes of the enrichment analysis were retained to plot the bar chart. The genomic alterations of 14-3-3 included missense mutation, truncating mutation, fusion, amplification, deep deletion, high mRNA expression and low mRNA expression.

### TIMER database analysis

TIMER analysis (http://www.cistrome.dfci.harvard.edu/TIMER/) was developed to assess immune cell infiltration in data from the TCGA dataset [42]. We investigated the correlations among 14-3-3 expression and the abundance of TIICs from gene expression profiles, including B cells, CD4+ T cells, CD8+ T cells, neutrophils, macrophages, and dendritic cells, as well as tumor purity. The scatterplots of 14-3-3 are displayed, showing Spearman’s correlation and statistical significance.

### NetworkAnalyst database analysis

The NetworkAnalyst database (https://www.network.analyst.ca/) is a visual analytics platform for comprehensive gene expression profiling and meta-analysis [43] that integrates cell-type or tissue-specific PPI networks, gene regulatory networks, and gene coexpression networks. We constructed a breast mammary tissue-specific PPI network to explore the potential mechanisms by which 14-3-3 isoforms participate in BrCa progression. GO and KEGG analyses were conducted in NetworkAnalyst by using the Function Explorer tool. Enrichment terms were considered statistically significant when the false discovery rate (FDR) was less than 0.05, and the top 10 terms of each analysis were retained to plot the bubble chart.

### Experimental validation

### IHC

The breast cancer tumor tissue microarray (TMA) HBre-Duc060CS-04 (30 cancer cases including tumor tissues and paired paracancerous tissues) was provided by Outdo Biotech (Shanghai, China). IHC was directly performed on the TMA. The primary antibodies used were as follows: anti-14-3-3 zeta (1: 100, Cat. A13370, ABclonal, Wuhan, China). Antibody staining was visualized with DAB and hematoxylin counterstain (ZSGB-Bio). The percentage of positively stained cells was scored on a scale of 0 to 4 as follows: 0 (<1%), 1 (1-10%), 2 (11-50%), and 3 (>50%). The staining intensity was scored from 0 to 3 as follows: 0 (negative), 1 (weak), 2 (moderate), and 3 (strong). The percentages of positive cells and staining intensities were then multiplied to generate an immunoreactivity score (IRS) for each case. IRS was performed without prior knowledge of clinical response. Immunostained sections were scanned using a microscope (Olympus Corporation, Tokyo, Japan).

### Cell culture

MDA-MB-231 cells were purchased from the Cell Bank of the Chinese Academy of Sciences (Shanghai, China). MDA-MB-231 cells were grown in Dulbecco’s modified Eagle’s medium (DMEM, high glucose) (HyClone, Thermo Scientific, Waltham, MA) supplemented with 10% (v/v) fetal bovine serum (FBS) (HyClone) in a humidified incubator at 37 °C with 5% CO_2_. MDA-MB-231 cells were verified to be mycoplasma negative monthly.

### Western blot analysis

MDA-MB-231 cancer cells were seeded into 6-well plates (6×10^5^ cells/well) and then transfected with the indicated siRNA: siRNA-14-3-3 zeta or siRNA control [27]. Forty-eight hours later, all cells were harvested and homogenized with lysis buffer. Total protein was separated by denatured 10% SDS-polyacrylamide gel electrophoresis. Western blot analysis was performed. The primary antibody for 14-3-3 zeta and GAPDH was purchased from ABclonal (Wuhan, China). Protein levels were normalized to GAPDH.

### Boyden chamber assays

Cell migration was assessed in a modified Boyden chamber (Costar, Corning, NY), in which the two chambers were separated by a polycarbonate membrane (pore diameter, 8.0 μm). Cells were grown to subconfluence in tissue culture plates, detached, centrifuged and rendered into single cell suspensions in serum-free culture medium supplemented with 5 mg/mL BSA. The suspensions containing 5×10^4^ cells were added to wells with a membrane placed at the bottom. Cells were allowed to migrate for 12 h at 37 °C. Thereafter, the medium was discarded; stationary cells were removed with a cotton-tipped applicator, and the membranes were cut out of the chamber and stained with 0.5% crystal violet. The response was evaluated under a light microscope by counting the number of cells that had migrated through the membrane.

### Wound healing assay

For wound healing analysis, MDA-MB-231 cells were seeded in 24-well plates (Costar, Corning, NY) and cultured to confluence. The cell monolayers were wounded by removing the culture insert and rinsed with PBS to remove cell debris. The images were acquired at 0 h and 24 h after migration using a Nikon optics microscope. The migratory area was calculated by measuring the distance from the edge of the wound closure.

### MTT assay

The MTT assay was conducted for cell viability determination. After 24 h of transfection, MDA-MB-231 cells were transferred to a 96-well plate (Costar, Corning, NY) at 5×10^3^ cells/well. Then, 20 μL MTT solution (5 μ mg/ml) was added to the plate at 24 h, 36 h, 48 h, and 72 h after being incubated at 37 °C, separately, and then the cells were incubated at 37 °C for 4 h. Then, 200 μL dimethyl sulfoxide was added to each well, and then the cell optical density of each group at 570 nm absorbance was determined via a spectrophotometer.

### Statistical analysis

All statistical analyses were conducted on the bioinformatics database online. The t-test was utilized to determine the statistical significance between groups with different expression levels of 14-3-3 isoforms. Kaplan-Meier curves were used to compare the survival time differences. Spearman’s correlation test was applied to evaluate associations between 14-3-3 expression and TIIC infiltration as well as tumor purity. Most of the data obtained from the experiment were analyzed by Student’s t-tests. For all analyses, differences were considered statistically significant if the p*-*values were less than 0.05.

## Supplementary Material

Supplementary Table 1
