# Correction for: Characterization of the expression and prognostic value of 14-3-3 isoforms in breast cancer

**DOI:** 10.18632/aging.204272

**Published:** 2022-08-30

**Authors:** Jie Mei, Yan Liu, Rui Xu, Leiyu Hao, An Qin, Chunqiang Chu, Yichao Zhu, Xiao Liu

**Affiliations:** 1 Department of Oncology, Wuxi People’s Hospital Affiliated to Nanjing Medical University, Wuxi 214023, Jiangsu, P.R. China; 2 Department of Physiology, Nanjing Medical University, Nanjing 211166, Jiangsu, P.R. China; 3 Department of General Surgery, Wuxi People’s Hospital Affiliated to Nanjing Medical University, Wuxi 214023, Jiangsu, P.R. China; 4 State Key Laboratory of Reproductive Medicine, Nanjing Medical University, Nanjing 211166, Jiangsu, P.R. China

**This article has been corrected: **The authors noticed an error in **Figure 2**. As a result of its misplacement, the graph in panel **2A**, which should show the gene expression data for 14-3-3 zeta, was the same as the graph in panel **2A**, which shows the gene expression data for 14-3-3 theta. The authors corrected panel **2A ** for 14-3-3 zeta in **Figure 2** by using the correct graph from the original sets of experiments. The authors stated that this alteration does not affect the results or conclusions of this work and apologized for any inconvenience caused.

New **Figure 2** is presented below.

**Figure 2:**
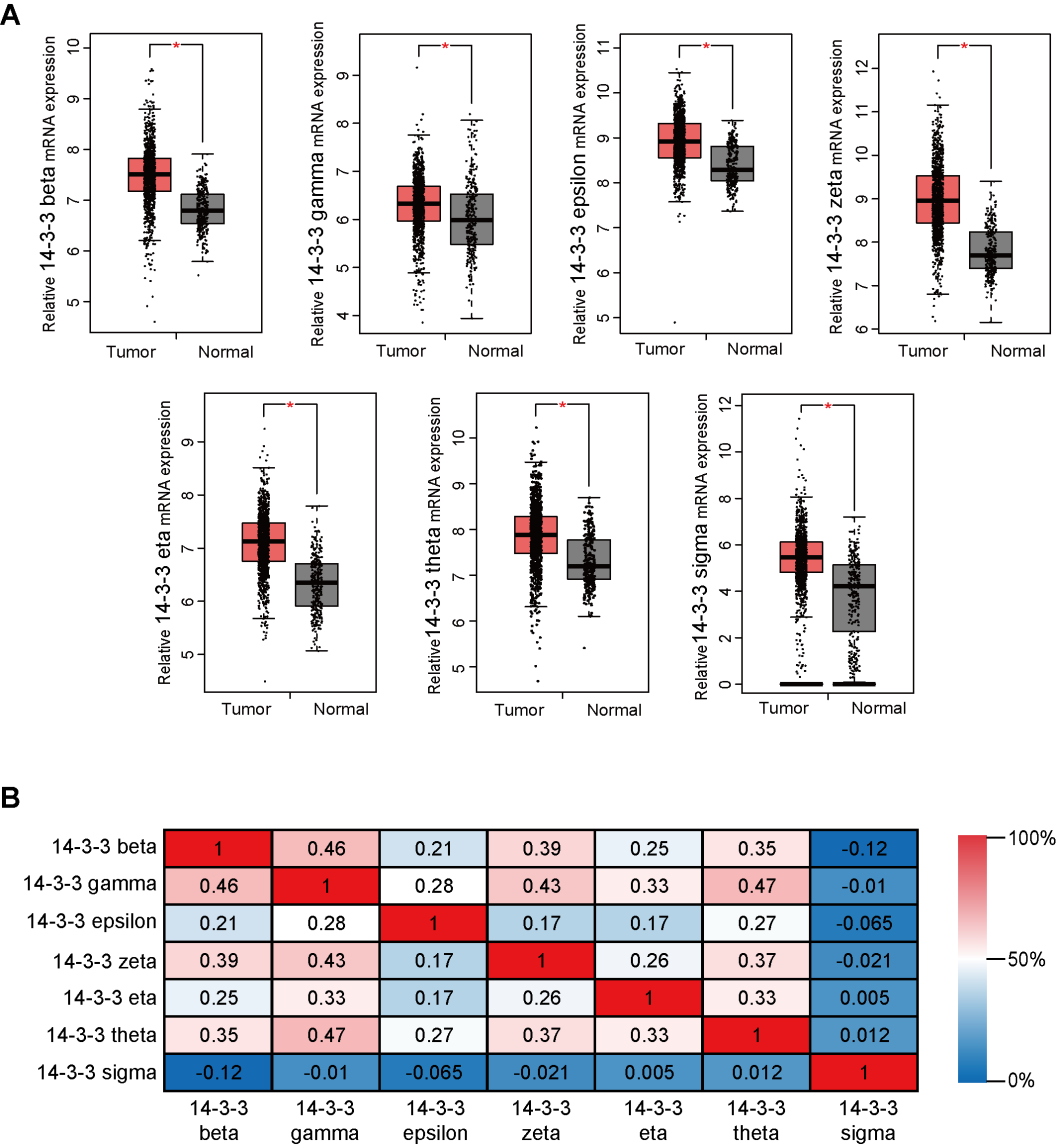
**Transcriptional levels of 14-3-3 in BrCa tissues.** (**A**) Box plots derived from gene expression data in GEPIA comparing the expression of 14-3-3 in BrCa and normal tissues. The p-value was set up at 0.05. (**B**) The Pearson correlation coefficients between 14-3-3 isoforms.

